# STRENGTHS AND LIMITATIONS OF USING DIFFERENT SENSOR TECHNOLOGIES TO ASSESS CLINICAL OUTCOMES IN DYADIC CARE

**DOI:** 10.1093/geroni/igae098.1412

**Published:** 2024-12-31

**Authors:** Neil Thomas, Bahareh Chimehi, Julien Lariviere-Chartier, Frank Knoefel, Zachary Beattie, Joel Steele, Lyndsey Miller, Bruce Wallace

**Affiliations:** Bruyere Research Institute, Ottawa, Ontario, Canada; Carleton University, Ottawa, Ontario, Canada; Bruyere Research Institute, Ottawa, Ontario, Canada; Bruyere Research Institute, Ottawa, Ontario, Canada; Oregon Health & Science University, Portland, Oregon, United States; University of North Dakota, Grand Forks, North Dakota, United States; Oregon Health & Science University, Portland, Oregon, United States; Carleton University, Ottawa, Ontario, Canada

## Abstract

Home sensor and smart home technologies offer the potential to provide important objective information on daily activities, functional abilities, and caregiving tasks for individuals with cognitive impairment and care partners. Combining information from multiple sensors, such as a bedmat, wearable, and motion sensors, can deliver more informative data on dyadic interactions during nighttime activity (e.g., room location and which partners are out of bed in addition to sleep measures). Preliminary work from a study developing a digital signature of caregiver burden using data from multiple sensors in a home technology platform is presented. Using data from two different sensors provided additional information on nighttime behaviors related to care partner burden level in a sample of 47 dyads (care partner and individual with dementia or mild cognitive impairment). Estimated sleep duration calculated from an under the mattress bedmat and activity monitoring wristwatch showed a low concordance (r=0.36). However, an additional 9156 nights were collected from the union of the two sensors as compared to the wristwatch (29318 nights) or bedmat (15704 nights) alone. Benefits to using multiple sensors include the ability to expand the amount of information collected on a dyad and the locations where information can be collected (e.g., sleep occurring outside of the bedroom). Challenges include determining the more accurate estimate when there is discordance between different sensor types and evaluating the accuracy of new or updated sensors. Additional work is needed to determine which sensor or combination of sensors is optimal at approximating a clinical outcome of interest.

